# Spectrum and Distribution of Dermatological Conditions Among University Students Attending a University Health Center in Southern Jordan: A Retrospective Study

**DOI:** 10.1155/drp/9651359

**Published:** 2026-11-08

**Authors:** Nedal Alnawaiseh, Khitam Al-Refu, Fadi Sawaqed

**Affiliations:** ^1^ Faculty of Medicine Mutah University Alkarak Mutah 61710 Jordan mutah.edu.jo; ^2^ Faculty of Medicine Mutah University P.O. Box 5, Alkarak Mutah 61710 Jordan mutah.edu.jo

**Keywords:** acne vulgaris, dermatology, Jordan, retrospective study, skin diseases, telogen effluvium, university students

## Abstract

**Background:**

Dermatological conditions are among the most common reasons for outpatient consultation worldwide. University students represent a distinct population whose dermatological health may be influenced by age‐related hormonal changes, academic stress, lifestyle changes, and environmental exposures. However, clinic‐based data describing dermatological conditions among university students in Jordan and the broader Middle East remain limited. This study aimed to describe the spectrum and clinic‐based distribution of dermatological conditions among undergraduate students attending the dermatology clinic of a university health center in Southern Jordan.

**Methods:**

A retrospective cross‐sectional study was conducted at the Dermatology Clinic of the University Health Center of Mutah University, Al‐Karak, Southern Jordan, covering a three‐year period from January 2023 to December 2025. All undergraduate students aged 18–25 years with a documented dermatological diagnosis during the study period were included. Data collected included age, gender, and primary dermatological diagnosis. Diagnoses were classified into major categories and ranked by frequency. Descriptive statistics were used for data analysis.

**Results:**

A total of 3782 undergraduate students were included, comprising 2021 females (53.4%) and 1761 males (46.6%), with a mean age of 21.2 ± 1.9 years. Acne vulgaris was the most frequently recorded condition (*n* = 1230; 32.5%), followed by hair disorders excluding alopecia areata (*n* = 519; 13.7%), eczema and dermatitis (*n* = 500; 13.2%), superficial fungal infections (*n* = 401; 10.6%), viral infections including warts (*n* = 285; 7.5%), pigmentary disorders excluding vitiligo (*n* = 204; 5.4%), rosacea (*n* = 148; 3.9%), urticaria (*n* = 134; 3.5%), bacterial infections (*n* = 94; 2.5%), alopecia areata (*n* = 72; 1.9%), and vitiligo (*n* = 68; 1.8%). No sexually transmitted dermatological conditions were documented in the reviewed clinic records.

**Conclusion:**

Acne vulgaris, hair disorders, and inflammatory dermatoses are the predominant dermatological conditions among university students in Southern Jordan, consistent with regional and global epidemiological trends. These findings highlight the importance of accessible dermatological services within university health settings and may inform targeted preventive and clinical strategies for this population.

## 1. Introduction

Skin diseases are among the most common health problems worldwide and represent a significant cause of morbidity and reduced quality of life across different populations. Epidemiological studies indicate that dermatological conditions account for a substantial proportion of outpatient consultations in both primary care and specialized clinics [1]. Although many skin disorders are not life‐threatening, their chronic nature, visibility, and recurrent course can significantly affect physical comfort, psychological well‐being, and social functioning [2].

The distribution and prevalence of dermatological diseases vary according to several factors, including age, gender, geographic location, climate, and socioeconomic conditions [3]. Young adults, particularly in Mutah university, which is located in Alkarak province, Jordan, feature a distinctive socioacademic environment divided into military and civilian wings. It hosts over 20,000 students, including a diverse international community, and emphasizes academic excellence, leadership, and sustainability. The atmosphere integrates traditional values, civic responsibility, and modern research, which represent a unique population group with distinct dermatological patterns. This age group commonly experiences conditions such as acne vulgaris, fungal infections, contact dermatitis, and hair disorders, which may be influenced by hormonal changes, lifestyle habits, stress, and environmental exposure [4]. University life is often associated with lifestyle modifications such as irregular sleep patterns, dietary changes, psychological stress, and increased social interaction, all of which may contribute to the development or exacerbation of certain skin conditions [5].

In addition to physical symptoms, dermatological diseases may have considerable psychosocial consequences among young adults. Skin conditions affecting visible areas such as the face or scalp can negatively influence self‐esteem, social confidence, and interpersonal relationships, particularly in environments where appearance plays an important role in social identity and peer interaction [6]. Previous studies conducted in different regions have shown that acne, eczema, and other common dermatoses can significantly affect the quality of life of university students and young adults [7].

Despite the high proportion of dermatological disorders in young populations, limited epidemiological data are available regarding the pattern of skin diseases among university students in many Middle Eastern countries, including Jordan. Understanding the distribution of dermatologic conditions in this population is important for planning preventive strategies, improving clinical services, and addressing potential public health concerns [8]. Southern Jordan has distinctive environmental and demographic features, most notably a predominantly arid desert climate, which may shape the pattern of dermatological diseases in the region. Despite this, epidemiological data from this part of the country remain scarce. Moreover, no prior large‐scale study exists specifically in Southern Jordan. So, the importance of studying dermatological disease patterns among university students in Southern Jordan has also been further emphasized.

Therefore, the present study aimed to describe the spectrum and clinic‐based distribution of dermatological conditions among undergraduate students attending the dermatology clinic of a university health center in Southern Jordan. The findings are intended to inform the planning of dermatological services within university healthcare settings and should not be interpreted as population‐level prevalence estimates.

## 2. Methods

### 2.1. Study Design and Setting

This retrospective cross‐sectional study was conducted at the University Health Center of Mutah University in Al‐Karak, Southern Jordan. The center provides primary medical services for university students as well as employees and their family members. For the purpose of this study, only undergraduate students attending the dermatology clinic were included in the analysis.

### 2.2. Study Period and Population

The source population comprised undergraduate students who attended the dermatology clinic at the University Health Center of Mutah University between January 2023 and December 2025.

### 2.3. Inclusion and Exclusion Criteria

Students were eligible for inclusion if they were undergraduate students aged 18–25 years and had a dermatological diagnosis documented by a board‐certified dermatologist during the study period. The individual student was the unit of analysis. Each student was identified using a unique patient code and included only once, based on the index consultation, defined as the first eligible dermatology consultation recorded during the study period. Follow‐up visits and repeated consultations were excluded from the frequency calculations. University employees, family members of staff, postgraduate students, and individuals with incomplete medical records were excluded.

## 3. Data Collection

Data were extracted retrospectively from paper‐based medical records. The collected variables included age, sex, date of consultation, and primary dermatological diagnosis. When more than one dermatological condition was documented for the same student, the student was classified according to the primary diagnosis responsible for the index consultation. Secondary or concurrent diagnoses were not counted separately in the main frequency analysis.

### 3.1. Classification of Dermatological Conditions

Dermatological diagnoses were assigned to mutually exclusive categories according to the primary diagnosis recorded at the index consultation. Hair disorders included acute and chronic telogen effluvium, androgenetic alopecia, and traction alopecia. Alopecia areata was excluded from this category and analyzed separately because it is an immune‐mediated hair‐loss disorder and was recorded as an independent diagnostic category in the dataset. Therefore, the 72 cases of alopecia areata were not included in the 519 cases classified as other hair disorders.

Eczema and dermatitis included atopic dermatitis, contact dermatitis, and seborrheic dermatitis. Superficial fungal infections included tinea corporis and pityriasis versicolor. Pigmentary disorders excluding vitiligo included melasma and postinflammatory hyperpigmentation. Vitiligo was analyzed as a separate diagnostic category.

The variables available for analysis were age, sex, date of consultation, and primary dermatological diagnosis. Information on disease severity, duration, treatment, nationality, military or civilian status, socioeconomic characteristics, psychological stress, dietary habits, hormonal influences, and other lifestyle characteristics was not consistently documented and was therefore not included in the analysis.

For the analysis, dermatological conditions were categorized according to their clinical diagnoses and epidemiological groupings. The frequency and relative distribution of each condition among clinic attendees were calculated, and the conditions were ranked according to their frequency of occurrence. Because this was a clinic‐based study, the reported proportions represent the distribution of diagnoses among students who attended the dermatology clinic and not the prevalence of these conditions among the overall university student population.

### 3.2. Statistical Analysis

Data were analyzed using IBM SPSS Statistics Version 29. Continuous variables were summarized using the mean and standard deviation. Categorical variables were summarized using frequencies and percentages. Dermatological conditions were ranked according to their proportion within the study population.

### 3.3. Ethical Considerations

The study protocol was reviewed and approved by the Institutional Review Board of Mutah University (Ref. No: 65023). Due to the retrospective nature of the study and the use of anonymized archival data, the requirement for informed consent was waived by the ethics committee. All procedures were conducted in accordance with the Declaration of Helsinki.

## 4. Results

### 4.1. Characteristics of the Study Population

During the three‐year study period from January 2023 to December 2025, dermatology consultations at the University Health Center of Mutah University were reviewed. The dermatology clinic receives approximately 35 patients weekly throughout the year. For the purpose of this study, only undergraduate students aged 18–25 years were included in the analysis.

A total of 3782 undergraduate students aged 18–25 years were included. The study population comprised 2021 females (53.4%) and 1761 males (46.6%), with a mean age of 21.2 ± 1.9 years (Table 1).

**TABLE 1:** Demographic characteristics among students attending the university dermatology clinic.

Variable	Number (%)
Total participants	3782 (100%)
Male	1761 (46.6%)
Female	2021 (53.4%)
Mean age of the participants	21.2 ± 1.9 years

### 4.2. Overall Distribution of Dermatological Conditions

As shown in Table 2, acne vulgaris was diagnosed in 1230 students (32.5%). Detailed information regarding acne severity, duration, and treatment was not consistently available in the reviewed records and was, therefore, not included in the analysis.

**TABLE 2:** Distribution of dermatological conditions among students attending the university dermatology clinic.

Disease	Number of cases	Percentage (%)
1. Acne vulgaris	1230	32.5
2. Hair disorders excluding alopecia areata	519	13.7
3. Eczema/dermatitis	500	13.2
4. Fungal infections	401	10.6
5. Viral infections (warts)	285	7.5
6. Pigmentary disorders	204	5.4
7. Rosacea	148	3.9
8. Urticaria	134	3.5
9. Bacterial infections	94	2.5
10. Alopecia areata	72	1.9
11. Vitiligo	68	1.8
12. Psoriasis	52	1.4
13. Keratosis pilaris	45	1.2
14. Lichen planus	21	0.6
15. Morphea	9	0.2

*Note:* Percentages were calculated using the total number of included patients as the denominator (*N* = 3782). Diagnostic categories were mutually exclusive, and each patient was counted once according to the primary dermatological diagnosis recorded at the index consultation. The 72 cases of alopecia areata were analyzed separately and were not included in the 519 cases classified as other hair disorders. Similarly, the 68 cases of vitiligo were not included in the 204 cases classified as other pigmentary disorders.

Hair disorders excluding alopecia areata represented the second most frequently recorded diagnostic category and were identified in 519 patients (13.7%). This category included acute and chronic telogen effluvium, androgenetic alopecia, and traction alopecia.

Eczema and dermatitis constituted another major category and were diagnosed in 500 patients (13.2%), including atopic dermatitis, contact dermatitis, and seborrheic dermatitis.

Superficial fungal infections were observed in 401 patients (10.6%), most commonly tinea corporis and pityriasis versicolor.

Viral skin infections, particularly common warts, were diagnosed in 285 patients (7.5%).

Pigmentary disorders were identified in 204 patients (5.4%), including melasma and postinflammatory hyperpigmentation. Vitiligo alone accounted for 68 cases (1.8%).

Rosacea was diagnosed in 148 patients (3.9%), while urticaria (hives) was observed in 134 patients (3.5%).

Bacterial skin infections, including folliculitis and impetigo, were identified in 94 patients (2.5%).

Alopecia areata was analyzed separately from the other hair disorders and was diagnosed in 72 patients (1.9%). Lichen planus was recorded in 21 patients (0.6%).

Other less common dermatological conditions included psoriasis, diagnosed in 52 patients (1.4%), keratosis pilaris in 45 patients (1.2%), and localized scleroderma (morphea) in 9 patients (0.2%), as shown in Table 2.

No sexually transmitted dermatological conditions were documented in the reviewed records of undergraduate students during the study period. This observation refers only to the available university clinic records and should not be interpreted as evidence that such conditions were absent from the wider student population.

## 5. Discussion

This clinic‐based retrospective study identified acne vulgaris as the most frequently recorded dermatological condition among undergraduate students attending the university dermatology clinic, followed by hair disorders excluding alopecia areata, eczema and dermatitis, superficial fungal infections, and viral infections. These findings describe the distribution of diagnoses among students who sought dermatological care and should not be interpreted as prevalence estimates for the overall university student population.

The predominance of acne is consistent with previous research involving university students and young adults [4, 9]. A systematic review of medical students in the Middle East reported a high burden of acne in this population although differences in study design and case ascertainment limit direct numerical comparison with the present clinic‐based findings [10]. Similarly, a study of students attending a university clinic in Turkey identified acne as the leading dermatological condition [11]. The frequent presentation of acne in university health settings supports the importance of accessible assessment, appropriate treatment, and counseling for affected students.

Hair disorders excluding alopecia areata represented the second most frequently recorded diagnostic category. Hair loss may be particularly likely to prompt consultation among young adults because of its visibility and potential psychosocial effect. Different forms of hair loss may be associated with nutritional, hormonal, psychological, or other clinical factors [12]. However, these variables were not consistently available in the reviewed records and cannot explain the observed frequency of hair disorders in the present study. Alopecia areata was analyzed separately because it is an immune‐mediated disorder and was recorded as an independent diagnostic category in the dataset.

Eczema and dermatitis, superficial fungal infections, and viral infections also accounted for substantial proportions of clinic attendance. Similar diagnostic categories have been reported in dermatology clinics in other Middle Eastern settings, although their relative distributions vary between studies [13]. These differences may reflect variation in population age, referral pathways, clinical setting, diagnostic classification, climate, and healthcare‐seeking behavior. Direct comparisons should, therefore, be made cautiously because many regional studies included general dermatology populations rather than university students specifically. However, these factors were not measured directly in the present study and should be considered possible explanations rather than conclusions supported by the data.

The observed diagnostic distribution may partly reflect patterns of healthcare utilization. Visible, symptomatic, persistent, or psychologically distressing conditions, such as acne and hair loss, may be more likely to prompt dermatological consultation than mild or self‐limiting disorders. Therefore, the frequency ranking in this study may reflect both the occurrence of the conditions and the likelihood that affected students sought care. Because the study did not include students who did not attend the clinic or a nonuniversity comparison group, it cannot determine whether the university environment independently influenced the observed distribution.

No sexually transmitted dermatological conditions were documented in the reviewed clinic records. This observation should not be interpreted as evidence that such conditions were absent from the wider university population. Privacy concerns, social stigma, underdiagnosis, or the use of healthcare services outside the university health center may have affected their documentation.

The findings support the provision of accessible dermatological services within university health settings, particularly for acne, hair disorders, inflammatory dermatoses, and superficial infections. Relevant services may include early clinical assessment, appropriate treatment, education regarding skin care and infection prevention, and referral for psychological support when visible or chronic dermatological conditions affect students’ well‐being.

Potential contributing factors discussed in relation to previous research, including psychological stress, dietary habits, hormonal influences, environmental exposure, hygiene, and lifestyle characteristics, were not consistently available in the retrospective records. Accordingly, they are presented only as possible hypotheses and were not evaluated as determinants of the dermatological conditions recorded in this study.

### 5.1. Study Limitations

This study has several limitations. Its single‐center, clinic‐based retrospective design may have introduced selection bias because only students who sought care at the university dermatology clinic were included; therefore, the findings may not be generalizable to the overall university student population. The reviewed records did not consistently contain detailed information on disease severity, duration, treatment, clinical subtypes, or potential contributing factors such as psychological stress, dietary habits, hormonal influences, and other lifestyle characteristics. Consequently, the study was limited to describing the distribution of the primary dermatological diagnoses documented among clinic attendees and could not assess the determinants or clinical course of these conditions.

## 6. Conclusion

This clinic‐based retrospective study describes the spectrum and distribution of dermatological conditions among undergraduate students who attended a university dermatology clinic in Southern Jordan. Acne vulgaris, hair disorders, eczema and dermatitis, and superficial fungal infections were the most frequently recorded conditions. These findings may assist in planning dermatological services within university healthcare settings. However, they should not be interpreted as prevalence estimates for the overall university student population. Population‐based and multicenter studies are needed to determine the prevalence and determinants of dermatological conditions among university students in Jordan.

## Funding

No external funding was received for this study.

## Disclosure

This manuscript has not been published previously and is not under consideration elsewhere.

All authors have read and approved the final manuscript.

## Ethics Statement

The study was conducted in accordance with the Declaration of Helsinki and received ethical approval from the Institutional Review Board of Mutah University.

## Conflicts of Interest

The authors declare no conflicts of interest.

## Data Availability

The data that support the findings of this study are available on request from the corresponding author. The data are not publicly available due to privacy or ethical restrictions.

## References

[1] Hay R. J. , Johns N. E. , Williams H. C. et al., The Global Burden of Skin Disease in 2010: An Analysis of the Prevalence and Impact of Skin Conditions, Journal of Investigative Dermatology. (2014) 134, no. 6, 1527–1534, 10.1038/jid.2013.446. 24166134

[2] Karimkhani C. , Dellavalle R. P. , Coffeng L. E. et al., Global Skin Disease Morbidity and Mortality: An Update From the Global Burden of Disease Study, JAMA Dermatology. (2017) 153, no. 5, 406–412, 10.1001/jamadermatol.2016.5538. PMC5817488 28249066

[3] Bickers D. R. , Lim H. W. , Margolis D. et al., The Burden of Skin Diseases: 2004 Report on the Burden of Skin Diseases in the United States, Journal of the American Academy of Dermatology. (2006) 55, no. 3, 490–500. 10.1016/j.jaad.2006.05.048 16908356

[4] Tan J. K. and Bhate K. , A Global Perspective on the Epidemiology of Acne, British Journal of Dermatology. (2015) 172, no. Suppl 1, 3–12, 10.1111/bjd.13462. 25597339

[5] Al-Shidhani A. , Al-Rashdi S. , Al-Habsi H. , and Rizvi S. , Impact of Acne on Quality of Life of Students at Sultan Qaboos University, Oman Medical Journal. (2015) 30, no. 1, 42–47, 10.5001/omj.2015.08. PMC4371463 25830000

[6] Dalgard F. J. , Gieler U. , Tomas-Aragones L. et al., The Psychological Burden of Skin Diseases: A Cross-Sectional Multicenter Study Among Dermatological Out-Patients in 13 European Countries, Journal of Investigative Dermatology. (2015) 135, no. 4, 984–991, 10.1038/jid.2014.530. PMC4378256 25521458

[7] Al Robaee A. A. , Assessment of General Health and Quality of Life in Patients With Acne Using a Validated Generic Questionnaire, Acta Dermatovenerologica Alpina Pannonica et Adriatica. (2009) 18, no. 4, 157–164. 20043053

[8] Seth D. , Cheldize K. , Brown D. , and Freeman E. F. , Global Burden of Skin Disease: Inequities and Innovations, Current Dermatology Reports. (2017) 6, no. 3, 204–210, 10.1007/s13671-017-0192-7. PMC5718374 29226027

[9] Lynn D. D. , Umari T. , Dunnick C. A. , and Dellavalle R. P. , The Epidemiology of Acne Vulgaris in Late Adolescence, Adolescent Health, Medicine and Therapeutics. (2016) 7, 13–25, 10.2147/ahmt.s55832. PMC4769025 26955297

[10] Alzahrani L. A. , Alfurayh N. A. , Jadaan N. S. , Alqahtani R. S. , and Alqahtani B. A. , Impact of Stress on the Prevalence of Acne Among Medical Students in the Middle East: A Systematic Review, Cureus. (2025) 17, no. 8, 10.7759/cureus.90815. PMC12452794 40988809

[11] Ertam I. , Babur Y. , Unal I. , and Alper S. , The Frequency of Skin Diseases Among Students in a University Clinic, Skinmed. (2010) 8, no. 5, 261–263. 21137634

[12] Almohanna H. M. , Ahmed A. A. , Tsatalis J. P. , and Tosti A. , The Role of Vitamins and Minerals in Hair Loss, Dermatologic Therapy. (2019) 9, no. 1, 51–70, 10.1007/s13555-018-0278-6. PMC6380979 30547302

[13] Krishnan S. and Almheiri K. , Pattern of Skin Diseases at a Dermatology Center: A Retrospective Study, Cureus. (2024) 16, no. 7, 10.7759/cureus.65259. PMC11343481 39184705

[14] Al Shobaili H. A. , The Pattern of Skin Diseases in the Qassim Region of Saudi Arabia: What the Primary Care Physician Should Know, Annals of Saudi Medicine. (2010) 30, no. 6, 448–453, 10.4103/0256-4947.72263. PMC2994160 21060156

[15] Alshamrani H. M. , Alsolami M. A. , Alshehri A. M. et al., Pattern of Skin Diseases in a University Hospital in Jeddah, Saudi Arabia: Age and Sex Distribution, Annals of Saudi Medicine. (2019) 39, no. 1, 22–28, 10.5144/0256-4947.2019.22. PMC6464678 30712047

